# Comprehensive analysis of the percentage of surface receptors and cytotoxic granules positive natural killer cells in patients with pancreatic cancer, gastric cancer, and colorectal cancer

**DOI:** 10.1186/1479-5876-11-262

**Published:** 2013-10-20

**Authors:** Yun-Peng Peng, Yi Zhu, Jing-Jing Zhang, Ze-Kuan Xu, Zhu-Yin Qian, Cun-Cai Dai, Kui-Rong Jiang, Jun-Li Wu, Wen-Tao Gao, Qiang Li, Qing Du, Yi Miao

**Affiliations:** 1 Department of General Surgery, The first Affiliated Hospital of Nanjing Medical University, 300 Guangzhou Road, Nanjing 210029, People’s Republic of China; 2 Jiangsu Province Academy of Clinical Medicine, Institute of Tumor Biology, 300 Guangzhou Road, Nanjing 210029, People’s Republic of China

**Keywords:** Cytotoxic granules, Digestive malignancies, NK cells, Surface receptors

## Abstract

**Background:**

Digestive malignancies, especially pancreatic cancer (PC), gastric cancer (GC), and colorectal cancer (CRC), still occur at persistently high rates, and disease progression in these cancers has been associated with tumor immunosurveillance escape. Natural killer (NK) cell dysfunction may be responsible for this phenomenon, however, the exact relationship between tumor immunosurveillance escape in digestive malignancies and NK cell dysfunction remains unclear.

**Methods:**

Percentage of the surface receptors NKG2A, KIR3DL1, NKG2D, NKp30, NKp44, NKp46, and DNAM-1, as well as the cytotoxic granules perforin and granzyme B positive NK cells were determined in patients with pancreatic cancer (n = 31), gastric cancer (n = 31), and CRC (n = 32) prior to surgery and healthy controls (n = 31) by multicolor flow cytometry. Independent *t*-tests or Mann-Whitney U-tests were used to compare the differences between the patient and healthy control groups, as well as the differences between patients with different pathologic features of cancer.

**Results:**

Percentage of NKG2D, NKp30, NKp46, and perforin positive NK cells was significantly down-regulated in patients with PC compared to healthy controls, as well as GC and CRC; reduced levels of these molecules was associated with indicators of disease progression in each malignancy (such as histological grade, depth of invasion, lymph node metastasis). On the contrary, percentage of KIR3DL1 positive NK cells was significantly increased in patients with PC, as well as GC and CRC, but was not associated with any indicators of disease progression.

**Conclusions:**

Altered percentage of surface receptors and cytotoxic granules positive NK cells may play a vital role in tumor immunosurveillance escape by inducing NK cell dysfunction in patients with PC, GC, and CRC.

## Background

Pancreatic cancer, gastric cancer and colorectal cancer are the most common digestive malignancies and have relatively high incidences. Pancreatic cancer is characterized by a low rate of early diagnosis and many tumors are unresectable [1], with a 5-year survival rate of only 6% [2] leading to a persistently high rate of mortality [3]. Colorectal cancer and gastric cancer are the third and fourth most common cancers worldwide, respectively, and are among the leading causes of cancer-related deaths [1]. In humans, the progression of certain malignancies is associated with the immune function of certain lymphocytes, such as natural killer (NK) cells. NK cells are CD16- and/or CD56-positive, and represent the first line of immune defense against transformed malignant cells [4].

When infection or malignancy occur, circulating NK cells become activated by cytokines and infiltrate into the affected tissues containing pathogen-infected or transformed malignant cells [5]. The direct cytotoxic effects of NK cells are determined by their expression of surface receptors and cytotoxic granules. NK cell dysfunction is observed in patients with certain types of cancer; therefore, surface receptors and cytotoxic granules are an important area of cancer research.

The natural cytotoxicity receptors (NCRs) NKp30, NKp44, and NKp46 are expressed on NK cells, as well as T cells and NK-like cells [6-9], and mediate NK cell activation during the process of natural cytotoxicity. Killer cell lectin-like receptor subfamily K, member 1 (NKG2D), a C-type lectin-like protein, is an activating receptor expressed on NK cells and also gamma-delta T cells, natural killer T (NKT) cells and other types of immune cells [10]. NKG2D is required for the ability of NK cells to lyse harmful cells [11,12]. NK cells also express other activating receptors including DNAX accessory molecule-1 (DNAM-1) which binds to two well-characterized ligands (nectin-2 and the poliovirus receptor) and exerts similar effects to NKG2D [13]. Killer cell lectin-like receptor subfamily C, member 1 (CD94/NKG2A-B), killer cell lectin-like receptor subfamily C, member 2 (CD94/NKG2C-E) and the killer immunoglobulin-like receptors (KIRs) are described as inhibitory receptors, which are important for the education of NK cells and NK-induced cytotoxicity through interacting with the major histocompatibility complex (MHC) class I allotype [14]. The cytotoxic granules perforin and granzyme B are intracellular molecules present in a number of lymphocytes, including NK cells. Perforin is required for the ability of granzyme B to promote apoptosis in target cells [15,16]. NK cells express high levels of perforin and granzyme B, and the expression levels of these molecules are closely associated with the cytotoxicity of NK cells [17].

## Methods

### Patients and healthy controls

Patients diagnosed with PC (*n* = 31), GC (*n* = 31), or CRC (*n* = 32) who were treated at Jiangsu Province Hospital were enrolled in this study. All patients had only received positive preoperative preparation and had not undergone radiotherapy, chemotherapy or any other therapeutic strategies prior to surgery. The main clinicopathological features of the patient cohorts are shown in Table 1. All peripheral blood samples were collected from the patients before surgery, and peripheral blood samples from 31 healthy control individuals were provided by Jiangsu Province Blood Center. This study was approved by the Ethics Committee of the First Affiliated Hospital of Nanjing Medical University. Each of the patients and healthy control individuals gave informed consent.

**Table 1:** Clinicopathological features of the patients and healthy controls included in this study

**Clinicopathological characteristics**					
**Groups**		**Healthy controls**	**Pancreatic cancer**	**Gastric cancer**	**Colorectal cancer**
		**n = 31**	**n = 31**	**n = 31**	**n = 32**
**Gender**	Male	21(67.7%)	23(74.2%)	20(64.5%)	17(53.1%)
	Female	10(32.3%)	8(26.8%)	11(35.5%)	15(46.9%)
**Age**	Median age	53	64	61	60
	Range	35-57	34-76	35-82	40-82
AJCC Stage^*^	0		0	1(3.2%)	0
	I		0	7(22.6%)	1(3.1%)
	II		20(64.5%)	4(12.9%)	16(50.0%)
	III		0	17(54.8%)	14(43.8%)
	IV		11(35.5%)	2(6.5%)	1(3.1%)

^*^2010 American Joint Committee on Cancer (AJCC).

### Reagents

The anti-human CD3-FITC/CD16 + 56-PE mixed antibody was obtained from Beckman Coulter (Brea, CA, USA). The anti-human CD3-FITC, CD16-PE/Cy7, CD56-PE/Cy7, NKG2D-PE/Cy7, NKp44-APC, NKp46-PE/Cy7, NKp30-APC, KIR3DL1-PE, DNAM-1-Alexa Fluor 647, and perforin-PerCP/Cy5.5 antibodies, and the RBC Lysis Buffer, Fixation Buffer and Wash Buffer were purchased from Biolegend (San Diego, CA, USA), as well as FITC, PE, PE/Cy7, APC, PerCP, Alexa Fluor-647, and PerCP/Cy 5.5 mouse IgG1 antibodies. The anti-human NKG2A-PerCP and granzyme B-APC antibodies were obtained from R&D Systems (Minneapolis, MI, UAS). All antibodies were mouse monoclonal antibodies.

### Preparation of peripheral blood samples and flow cytometric analysis

Each peripheral blood sample (2 ml) was aliquoted into four tubes (100 μl per tube), which were labeled tube-1, tube-2, tube-3 and tube-4, respectively.

Peripheral blood samples of tube-1, tube-2 and tube-3 were stained to detect surface receptors as follows. Firstly, to identify NK cells, anti-human CD3-FITC/CD16 + 56-PE mixed antibodies were added to tube-1 and tube-2. Anti-human CD3-FITC, CD16-PE/Cy7 and CD56-PE/Cy7 antibodies were added to tube-3. Secondly, anti-human NKG2D-PE/Cy7 and NKp44-APC antibodies were added to tube-1. Anti-human NKG2A-PerCP, NKp46-PE/Cy7 and NKp30-APC antibodies were added to tube-2. Anti-human KIR3DL1-PE and DNAM-1-Alexa Fluor-647 antibodies were added to tube-3. The three tubes were incubated in the dark at room temperature for 15-20 min. Then 2 ml RBC Lysis Buffer was added per tube. After incubating in the dark at room temperature for 15 min, the cells were washed twice with PBS.

Peripheral blood sample of Tube-4 was stained to detect cytotoxic granules as follows. Firstly, anti-human CD3-FITC/CD16 + 56-PE mixed antibodies were added to tube-4 to identify NK cells. After incubating in the dark at room temperature for 15-20 min, 2 ml RBC Lysis Buffer was added per tube, and the mixtures were incubated in the dark at room temperature for 15 min. Then the cells were washed twice with PBS and fixation Buffer (500 μl per tube) was added. The mixtures were incubated in the dark at room temperature for 20 min, and then the cells were washed twice with Wash Buffer. Lastly, anti-human perforin-PerCP/Cy5.5 and granzyme B-APC antibodies were added to tube-3. After incubating in the dark at room temperature for 15 min, the cells were washed twice with PBS.

### Flow cytometric analysis

According to cell physical characteristics, forward scatter (FSC) and side scatter (SSC), a cell subset located in left lower quadrant (PBMCs) was selected from total cell subset and defined as gating “A”. And then, according to cells staining, another cell subset which detected as CD3-/CD(16 + 56) + (NK cells) was selected from gating “A” and defined as gating “Q”. Further detections for surface receptors and cytotoxic granules were based on cells from gating “Q”. The whole detection for per tube would stop until getting 10000 cells from gating “Q”. Isotype control was applied in our study to exclude non-specific fluorescence using matched isotype monoclonal antibodies (FITC, PE, PE/Cy7, and APC mouse IgG1 antibodies for tube 1; FITC, PE, PE/Cy7, PerCP, and APC mouse IgG1 antibodies for tube 2; FITC, PE, PE/Cy7, and Alexa Fluor-647 antibodies for tube 3; FITC, PE, PerCP/Cy 5.5, and APC mouse IgG1 antibodies for tube 4). Data were detected by multicolor flow cytometry (Gallios, Beckman Coulter, Brea, CA, USA) and gallios software (Beckman Coulter, Brea, CA, USA), and analyzed by Kaluza software (Beckman Coulter, Brea, CA, USA).

### Statistical analysis

Independent *t*-tests were used to compare the differences between two groups when the two groups both accorded with normal distribution, otherwise Mann–Whitney U-tests were used. Independent *t*-tests and Mann–Whitney U-tests were performed using Statistical Product and Service Solutions 19.0 (SPSS 19.0) (SPSS Inc., Chicago, IL, USA). Data were expressed as means ± standard deviations (Mean ± SD). The level of statistical significance accepted was P < 0.05.

## Results

### Percentage of surface receptor and cytotoxic granule positive circulating NK cells

**Figure 1:**
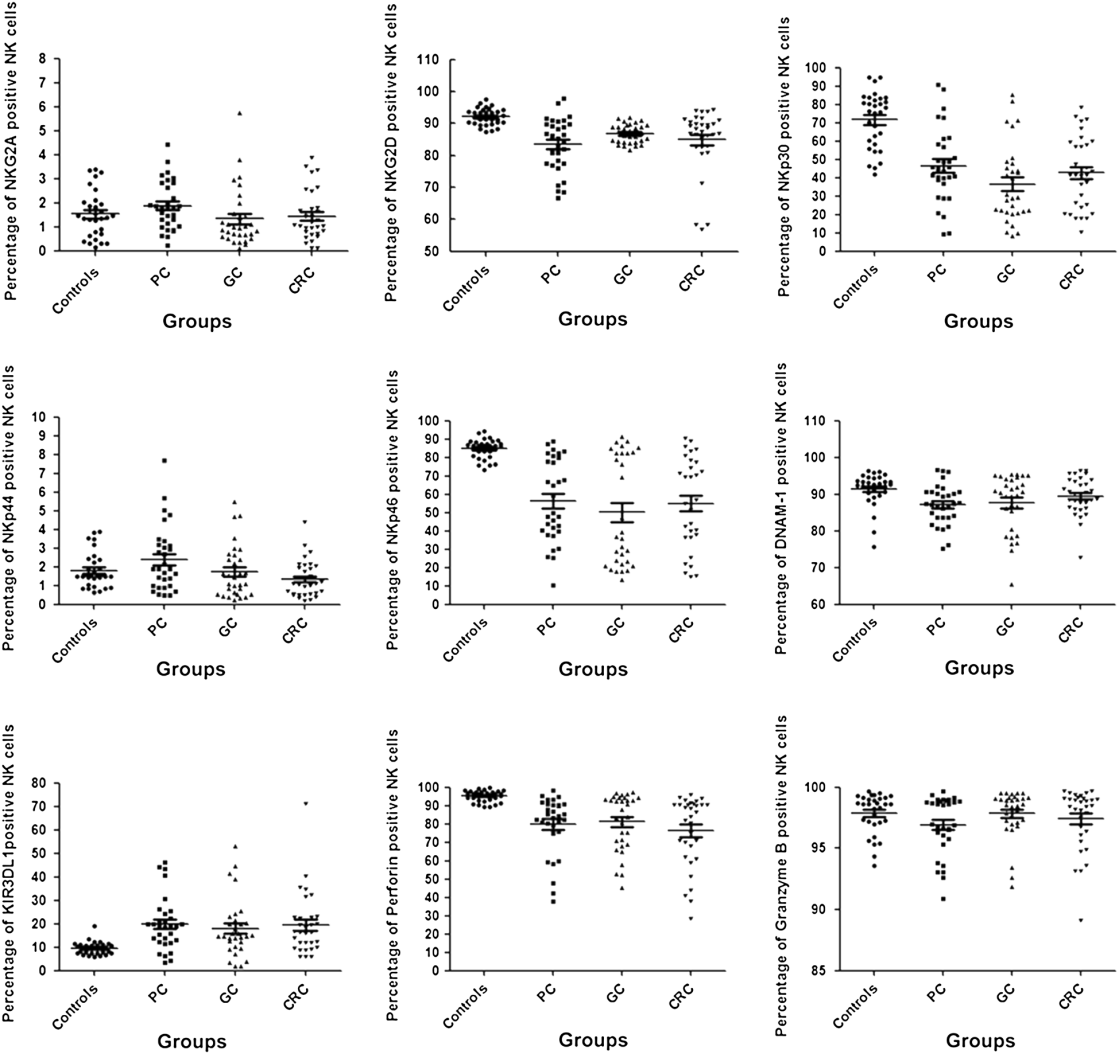
**Distribution of the percentage of surface receptor and cytotoxic granule positive circulating NK cells in healthy controls (Controls) and patients with pancreatic cancer (PC), gastric cancer (GC), and colorectal cancer (CRC).** The bar for each group was means and standard deviations (Mean and SD).

We determined the percentage of seven surface receptors positive circulating NK cells in both healthy controls and patients with PC, GC, and CRC by multicolor flow cytometry. The percentage of tested molecules positive circulating NK cells of the cancer patients and healthy controls are presented in Figure 1 and Table 2.

**Table 2:** Respective comparison of the percentage of surface receptors and cytotoxic granules positive NK cells in healthy controls and three kinds of cancer patients

	**Healthy controls**	**Pancreatic cancer**		**Gastric cancer**		**Colorectal cancer**	
	**%**	**%**	**P**	**%**	**P**	**%**	**P**
**NKG2A**	1.5 ± 1.0	1.9 ± 1.0	ns ^U^	1.3 ± 1.2	ns ^U^	1.4 ± 1.0	ns ^U^
**NKG2D**	92.1 ± 2.7	83.4 ± 8.4	< 0.001 ^T^	86.9 ± 2.9	< 0.001 ^T^	84.9 ± 10.0	< 0.01 ^U^
**NKp30**	71.8 ± 15.3	46.5 ± 20.2	< 0.001 ^U^	36.6 ± 21.3	< 0.001 ^U^	42.6 ± 19.4	< 0.001 ^U^
**NKp44**	1.8 ± 0.9	2.4 ± 1.7	ns ^U^	1.7 ± 1.4	ns ^U^	0.9 ± 1.3	ns ^U^
**NKp46**	84.8 ± 5.1	56.4 ± 22.2	< 0.001 ^U^	50.1 ± 29.5	< 0.001 ^U^	55.1 ± 24.0	< 0.001 ^U^
**DNAM-1**	91.4 ± 4.6	87.2 ± 5.5	< 0.01 ^U^	87.7 ± 7.8	ns ^U^	89.5 ± 5.1	ns ^U^
**KIR3DL1**	9.6 ± 2.7	19.8 ± 11.4	< 0.001 ^U^	17.9 ± 12.3	< 0.001 ^U^	19.5 ± 13.1	< 0.001 ^U^
**Granzyme B**	97.9 ± 1.6	96.9 ± 2.4	ns ^U^	97.8 ± 2.0	ns ^U^	97.4 ± 2.5	ns ^U^
**Perforin**	95.2 ± 3.0	79.9 ± 16.0	< 0.01 ^U^	81.2 ± 15.5	< 0.001 ^U^	76.3 ± 19.1	< 0.001 ^U^

^U^ represented Mann–Whitney U-tests and ^T^ represented independent t-tests. Data were expressed as means ± standard deviations (Mean ± SD).

Compared to the healthy controls, significantly decreased levels of activating receptors NKG2D, NKp30, NKp46, and DNAM-1 positive NK cells were observed in PC patients (*P* < 0.001, *P* < 0.001, *P* < 0.001, and *P* < 0.01, respectively); however, an significantly increased level of inhibitory receptor KIR3DL1 positive NK cells was observed in patients with PC (*P* < 0.001). In GC patients, the activating receptors NKG2D, NKp30, and NKp46 positive NK cells were also significantly down-regulated compared to the healthy controls (*P* < 0.001, *P* < 0.001, and *P* < 0.001, respectively); however, the inhibitory receptor KIR3DL1 positive NK cells was also significantly up-regulated compared to the healthy controls (*P* < 0.001). Furthermore, the levels of activating receptors NKG2D, NKp30, and NKp46 positive NK cells in CRC patients was significantly lower compared to healthy controls (*P* < 0.01, *P* < 0.001, and *P* < 0.001, respectively); however, the level of inhibitory receptor KIR3DL1 positive NK cells was also significantly higher compared to the healthy controls (*P* < 0.001).

We also determined the percentage of cytotoxic perforin and granzyme B positive circulating NK cells in both healthy controls and patients with PC, GC, and CRC (Figure 1 and Table 2). Respectively compared to the healthy controls, percentage of perforin positive NK cells was significantly lower in patients with PC, GC, and CRC (*P* < 0.01, *P* < 0.001, and *P* < 0.001, respectively). Percentage of granzyme B positive NK cells was at high levels in both the NK cells of the patients with cancer and the healthy controls.

### Altered percentage of NKG2D, NKp30, NKp46, KIR3DL1, and perforin positive NK cells correlate with disease progression

The correlations between the percentage of NKG2D, NKp30, NKp46, KIR3DL1, and perforin positive NK cells and the pathologic features of PC, GC, and CRC are respectively shown in Tables 3, 4 and 5.

**Table 3:** Association between the percentage of surface receptors and cytotoxic granules positive NK cells and the clinicopathological features of pancreatic cancer

**Pancreatic cancer**											
	**NO. of patients**	**NKG2D**		**NKp30**		**NKp46**		**Perforin**		**KIR3DL1**	
		**%**	**P**	**%**	**P**	**%**	**P**	**%**	**P**	**%**	**P**
**Distant metastasis**											
Absent	20	82.6 ± 9.5	ns ^T^	44.2 ± 19.3	ns ^T^	53.8 ± 23.4	ns ^T^	80.5 ± 16.8	ns ^U^	20.6 ± 12.1	ns ^U^
Present	11	85.5 ± 6.4		50.7 ± 22.1		60.9 ± 20.2		78.7 ± 15.0		18.4 ± 10.3	
**Non-metastatic Pancreatic cancer**											
	**NO. of patients**	**NKG2D**		**NKp30**		**NKp46**		**Perforin**		**KIR3DL1**	
		**%**	**P**	**%**	**P**	**%**	**P**	**%**	**P**	**%**	**P**
**Histological grade**											
Well/Moderately	9	85.4 ± 8.9	ns ^T^	46.2 ± 27.1	ns ^T^	71.4 ± 17.4	< 0.01 ^U^	85.3 ± 8.5	ns ^U^	23.2 ± 13.6	ns ^U^
Poorly	11	79.6 ± 9.1		40.6 ± 12.4		39.5 ± 17.1		76.5 ± 21.0		18.6 ± 10.8	
Depth of invasion^*^											
Tis/T1/T2	11	84.3 ± 7.6	ns ^T^	41.2 ± 18.2	ns ^T^	54.4 ± 22.6	ns ^T^	75.2 ± 19.1	ns ^U^	18.7 ± 10.4	ns ^T^
T3	9	79.7 ± 10.9		46.0 ± 22.9		53.2 ± 25.6		86.9 ± 11.6		23.0 ± 14.1	
**Lymph node metastasis**											
Absent	6	89.9 ± 5.6	< 0.05 ^T^	58.1 ± 19.0	< 0.05 ^T^	60.9 ± 22.6	ns ^T^	80.9 ± 21.7	ns ^U^	27.0 ± 15.5	ns ^T^
Present	14	78.9 ± 8.6		37.8 ± 17.7		50.8 ± 23.8		80.3 ± 15.3		17.9 ± 9.7	
**Blood vessel invasion**											
Absent	13	86.7 ± 6.7	< 0.01 ^T^	40.3 ± 22.7	ns ^U^	54.7 ± 24.9	ns ^U^	86.2 ± 12.9	< 0.05 ^U^	21.9 ± 14.4	ns ^U^
Present	7	73.9 ± 7.6		48.4 ± 13.7		52.2 ± 22.1		69.9 ± 19.1		18.3 ± 6.1	
**Nerve invasion**											
Absent	6	86.9 ± 5.7	ns ^T^	36.2 ± 22.6	ns ^U^	60.5 ± 27.6	ns ^U^	78.1 ± 16.1	ns ^U^	15.9 ± 5.6	ns ^T^
Present	14	80.2 ± 9.9		45.9 ± 19.0		51.0 ± 21.8		81.5 ± 17.6		22.7 ± 13.6	

^*^According to 2010 American Joint Committee on Cancer (AJCC), T4 pancreatic cancer invades celiac artery or superior mesenteric artery, and is unresectable, so T4 is not enrolled in depth of invasion for non-metastatic pancreatic cancer. Tis, T1, or T2 pancreatic cancer invades inside of the pancreas; T3 pancreatic primary cancer invades outside of the pancreas. ^U^ represented Mann–Whitney U-tests and ^T^ represented independent t-tests. Data were expressed as means ± standard deviations (Mean ± SD).

**Table 4:** Association between the percentage of surface receptors and cytotoxic granules positive NK cells and the clinicopathological features of gastric cancer

**Gastric cancer**											
	**NO. of patients**	**NKG2D**		**NKp30**		**NKp46**		**Perforin**		**KIR3DL1**	
		**%**	**P**	**%**	**P**	**%**	**P**	**%**	**P**	**%**	**P**
**Histological grade**											
Well/Moderately	12	89.0 ± 3.0	< 0.01 ^T^	45.1 ± 24.4	< 0.05 ^U^	56.3 ± 28.7	ns ^U^	88.5 ± 7.6	< 0.05 ^U^	15.3 ± 5.9	ns ^U^
Poorly	19	86.2 ± 2.5		31.2 ± 17.7		46.2 ± 30.1		76.5 ± 17.4		20.5 ± 14.3	
Depth of invasion^*^											
Tis/T1/T2/T3	10	87.8 ± 3.2	ns ^T^	48.0 ± 24.3	< 0.05 ^T^	54.2 ± 29.1	ns ^U^	85.5 ± 10.0	ns ^U^	21.4 ± 12.7	ns ^U^
T4	21	87.0 ± 2.9		31.1 ± 17.8		48.1 ± 29.1		79.1 ± 17.3		17.2 ± 11.6	
**Lymph node metastasis**											
Absent	11	89.3 ± 2.8	< 0.01 ^T^	48.2 ± 22.7	< 0.05 ^T^	57.6 ± 29.3	ns ^U^	88.5 ± 8.1	< 0.05 ^U^	15.1 ± 5.7	ns ^U^
Present	20	86.1 ± 2.5		30.1 ± 17.9		46.0 ± 29.5		77.1 ± 17.1		20.4 ± 14.0	
**Blood vessel invasion**											
Absent	17	88.3 ± 3.0	< 0.05 ^T^	38.0 ± 23.8	ns ^T^	49.0 ± 29.6	ns ^U^	84.5 ± 12.0	ns ^U^	18.3 ± 10.8	ns ^U^
Present	14	85.9 ± 2.5		34.8 ± 18.4		51.4 ± 30.5		77.1 ± 18.5		18.8 ± 13.6	

^*^According to 2010 American Joint Committee on Cancer (AJCC), Tis, T1, T2, or T3 gastric cancer invades inside of gastric serosa; T4 gastric cancer invades outside of gastric serosa or adjacent organs. ^U^ represented Mann–Whitney U-tests and ^T^ represented independent t-tests. Data were expressed as means ± standard deviations (Mean ± SD).

**Table 5:** Association between the percentage of surface receptors and cytotoxic granules positive NK cells and the clinicopathological features of colorectal cancer

**Colorectal cancer**											
	**NO. of patients**	**NKG2D**		**NKp30**		**NKp46**		**Perforin**		**KIR3DL1**	
		**%**	**P**	**%**	**P**	**%**	**P**	**%**	**P**	**%**	**P**
**Histological grade**											
Well/Moderately	20	89.0 ± 3.7	< 0.01 ^U^	39.5 ± 19.3	ns ^T^	56.0 ± 25.8	ns ^T^	83.9 ± 11.6	< 0.05 ^U^	20.1 ± 15.9	ns ^U^
Poorly	12	77.9 ± 13.3		47.8 ± 19.2		53.6 ± 21.8		63.5 ± 22.8		18.6 ± 6.6	
Depth of invasion^*^											
Tis/T1/T2/T3	7	88.9 ± 4.3	ns ^U^	57.0 ± 12.4	< 0.05 ^T^	71.4 ± 19.5	< 0.05 ^T^	87.7 ± 13.0	< 0.05 ^U^	20.7 ± 11.1	ns ^U^
T4	25	83.7 ± 10.9		38.6 ± 19.2		50.5 ± 23.5		73.1 ± 19.6		19.2 ± 13.8	
**Lymph node metastasis**											
Absent	18	89.3 ± 4.2	< 0.01 ^U^	39.3 ± 20.2	ns ^U^	63.8 ± 24.8	< 0.05 ^U^	86.2 ± 8.8	< 0.01 ^U^	21.2 ± 16.8	ns ^U^
Present	14	79.1 ± 12.4		46.9 ± 18.1		43.8 ± 18.1		63.5 ± 21.5		17.4 ± 5.5	
**Blood vessel invasion**											
Absent	25	86.5 ± 7.8	ns ^U^	42.0 ± 20.1	ns ^U^	53.9 ± 24.1	ns ^U^	77.8 ± 18.2	ns ^U^	19.0 ± 14.5	ns ^U^
Present	7	78.9 ± 15.0		44.6 ± 17.9		59.1 ± 25.2		70.7 ± 23.0		21.4 ± 6.3	
**Nerve invasion**											
Absent	27	84.0 ± 11.7	ns ^U^	40.9 ± 18.6	ns ^T^	55.7 ± 23.3	ns ^U^	75.8 ± 19.7	ns ^U^	20.8 ± 13.7	ns ^U^
Present	5	84.7 ± 3.1		51.8 ± 23.1		51.4 ± 30.4		78.8 ± 17.8		12.7 ± 6.5	

^*^According to 2010 American Joint Committee on Cancer (AJCC), Tis, T1, T2, or T3 colorectal cancer invades inside of colorectal serosa or non-peritonealized adjacent colorectal tissue; T4 gastric cancer invades outside of colorectal serosa or adjacent organs. ^U^ represents Mann–Whitney U-tests and ^T^ represents independent t-tests. Data were expressed as means ± standard deviations (Mean ± SD).

In pancreatic cancer, NKG2D, NKp30, NKp46, KIR3DL1, and perforin had no association with the presence of distant metastasis. In non-metastatic pancreatic cancer, the percentage of NKG2D and NKp30 positive NK cells were significantly decreased in patients with lymph node metastasis than patients without lymph node metastasis (both *P* < 0.05). The levels of NKG2D and perforin positive NK cells were significantly lower in patients with blood vessel invasion, compared to patients with non-metastatic pancreatic cancer who did not have blood vessel invasion (*P* < 0.05 and *P* < 0.01). NKp46 positive NK cells percentage also correlated closely with the histological grade in non-metastatic pancreatic cancer (*P* < 0.01).

In gastric cancer, the percentage of NKG2D, NKp30, and perforin positive NK cells were significantly lower in patients with lymph node metastasis than patients without lymph node metastasis (*P* < 0.01, *P* < 0.05 and *P* < 0.05, respectively). NKG2D positive NK cells were significantly down-regulated in patients with blood vessel invasion compared to patients without blood vessel invasion (*P* < 0.05). NKG2D, NKp30, and perforin positive NK cells were significantly higher levels in patients with gastric cancer who had well or moderately differentiated tumors, compared to those with poorly differentiated tumors (*P* < 0.01, *P* < 0.05, and *P* < 0.05, respectively). Moreover, the percentage of NKp30 positive NK cells correlated significantly with the depth of invasion in gastric cancer (*P* < 0.05).

In colorectal cancer, NKG2D, NKp46, and perforin positive NK cells were significantly lower levels in patients with lymph node metastasis compared to patients without lymph node metastasis (*P* < 0.01, *P* < 0.05, and *P* < 0.01). The percentage of NKp30, NKp46, and perforin positive NK cells correlated markedly with depth of invasion in CRC (all *P* < 0.05). The percentage of NKG2D and perforin positive NK cells correlated closely with histological grade in CRC (*P* < 0.01 and *P* < 0.05). None of the molecules tested were associated with blood vessel invasion or nerve invasion in CRC.

## Discussion

In this study, we quantified the percentage of several activating and inhibitory surface receptors positive circulating NK cells, as well as the cytotoxic granules perforin and granzyme B, in patients with PC, GC, and CRC. The balance between activating and inhibitory receptors has been shown to be a key factor which determines NK cell activity [18]. It has been demonstrated that NK-mediated anti-tumor immunity is frequently defective in patients with certain malignancies [19,20]. This study indicates that patients with PC, GC, and CRC have dysfunctional NK cells; therefore, NK cell dysfunction may be an important component of tumor escape from immunosurveillance in these cancers.

NKp30, NKp44, and NKp46 are the most well characterized NCRs. Our results show for the first time that the numbers of NKp30 and NKp46-positive NK cells were significantly reduced in almost all patients with PC, GC, and CRC, consistent with studies in other malignancies such as cervical cancer, breast cancer, and melanoma [21-23]. It has been reported that NCR-positive NK cells have the ability to kill harmful cells, such as transformed malignant cells and infected cells, and can also secret inflammatory cytokines such as interferon-γ (IFN-γ) and tumor necrosis factor-α (TNF-α) [24]. Accordingly, the lower number of cells expressing NKp30 and NKp46 may be partly responsible for the poor function of NK cells in patients with PC, GC, and CRC. The NCR-mediated interaction between NK cells and their target cells is ligand-dependent. Cellular heparin or heparin sulfate proteoglycans, which are expressed at high levels on cancer cells, are ligands for all NCRs [25], while natural killer cell cytotoxicity receptor 3 ligand 1 (B7-H6) and BCL2-associated athanogene 6 (BAT3) are specific ligands for NKp30 [26,27]. The specific ligands for NKp46 are associated with different cells, for example, Thr^225^ for some malignancy cells, Thr^125^ and Asn^216^ for human β cells [28]. It has been shown that NKp30 is blocked by exosomal and soluble BCL-2-associated athanogene-6 (BAG6) which are released by cancer cells; however, during infection with certain viruses, soluble influenza haemagglutinin (HA) and pp65 take similar effect to block NKp30 or NKp46. We suggest that such blocking effects may contribute to the downregulation of NKp30 and NKp46 [24] on NK cells in patients with PC, GC and CRC; however, the exact mechanisms require further research.

NKp30- and NKp46-mediated cytotoxicity of NK cells are not only linked to the elimination of cancer cells, but also to the eradication of bacterial and viral infection, and regulation of immune homeostasis [29]. Further analysis of our data revealed that the expression of NKp30 and NKp46 correlated with pathological stage and histological grade in patients with PC, GC and CRC, which indicates that NK cell dysfunction may participate in malignant progression in these tumor types.

NKG2D is an important activating receptor on NK cells. In patients with cancer, NKG2D generally binds specifically to killer cell lectin-like receptor subfamily K, member 1 ligands (NKG2DLs) expressed on transformed malignant cells, such as MHC class I-related molecules, MHC class I polypeptide-related sequence A/B (MICA/MICB), and UL16-binding protein (ULBP) [30]. The NKG2D-NKG2DL complex associates with the hematopoietic cell signal transducer (DAP10) adaptor protein and induces the cytotoxic effects of NK cells via the phosphatidylinositol-3 kinase (PI-3-K) pathway [4]. A number of soluble factors, such as TGF-β and L-kynurenine, which are secreted at high levels by malignant cells, are effective inhibitors of NKG2D-associated NK cell function [31,32]. However, our results indicate that the NKG2D-mediated interaction between NK cells and cancer cells is reduced during the development of PC, GC, and CRC, and similar results have previously been reported in pancreatic cancer, gastric cancer, and other types of cancer [20,33,34]. Additionally, Guerra et al. demonstrated that *NKG2D*-deficient mice exhibit defective tumor surveillance in models of spontaneous malignancy, which also supports our results [35]. It is noteworthy that not only down-regulation of NKG2D, but also the release of NKG2DLs from the surface of cancer cells may contribute to NK cell dysfunction and the progression of some types of cancer [36].

Furthermore, our results also suggest that reduced expression of NKG2D and perforin by NK cells correlated significantly with lymph node metastasis in PC, GC, and CRC, and also correlated with histological grade in gastric cancer and CRC. The view that perforin-dependent cytotoxicity is a crucial factor in NKG2D-mediated apoptosis [37] is confirmed by this study. In response to infection or cancer, the cytotoxic granule granzyme B associates with perforin in NK cells to form a complex which is ultimately released into the cytoplasm of the target cell and mediates the cytotoxic effects of NK cells [15,16]. Therefore, reduced expression of perforin by NK cells in patients with PC, GC, and CRC may form a crucial part of the mechanism of NK dysfunction in these cancers.

We also investigated NK cell inhibitory receptors in this study. KIR3DL1, a well-characterized killer immunoglobulin-like receptor, binds the specific ligand major histocompatibility complex, class I, Bw4 (HLA-Bw4) [38]. Our results show that the expression of KIR3DL1 by NK cells was significantly increased in patients with PC, GC, and CRC. However, this increase did not correlate significantly with any pathological feature. Al Omar et al. reported similar result in patients with kidney cancer and small-cell lung cancer, but not in patients with non-small-cell lung cancer and colon cancer [39]. Further research is required to determine the role of KIR3DL1 in different types of cancer.

## Conclusions

In conclusion, down-regulated percentage of the activating receptors NKp30, NKp46, and NKG2D positive NK cells, as well as the cytotoxic granule perforin, in patients with PC, GC, and CRC may indicate that patients with these digestive system cancers have dysfunctional NK cells. Additionally, the percentage of these molecules positive NK cells correlated with certain clinicopathological features. Thus, in certain malignancies, NK cell dysfunction may potentially promote the escape of malignant cells from immunosurveillance, and may also be a marker of poor prognosis. Further research is required to determine the exact mechanisms for why these molecules positive NK cells are down-regulated in patients with digestive system cancers; such research may contribute to immunotherapy strategies to improve NK cell function in patients with cancer.

## Abbreviations

B7-H6: Natural killer cell cytotoxicity receptor 3 ligand 1; BAT3/BAG6: BCL2-associated athanogene 6; CRC: Colorectal cancer; DAP10: Hematopoietic cell signal transducer; DNAM-1: DNAX accessory molecule-1; FSC: Forward scatter; GC: Gastric cancer; HA: Haemagglutinin; HLA-Bw4: Major histocompatibility complex, class I, Bw4; IFN-γ: Interferon-γ; KIR3DL1: Killer cell immunoglobulin-like receptor, three domains, long cytoplasmic tail, 1; MHC: Major histocompatibility complex; MICA/MICB: MHC class I polypeptide-related sequence A/B; NCRs: Natural cytotoxicity receptors; NK cells: Natural killer cells; NK cells: Natural killer T cells; NKG2A: Killer cell lectin-like receptor subfamily C, member 1-like; NKG2D: Killer cell lectin-like receptor subfamily K, member 1; NKG2DLs: Killer cell lectin-like receptor subfamily K, member 1 ligands; NKp30: Natural cytotoxicity triggering receptor 3; NKp44: Natural cytotoxicity triggering receptor 2; NKp46: Natural cytotoxicity triggering receptor 1; PC: Pancreatic cancer; PI-3-K: Phosphatidylinositol-3 kinase; SD: Standard deviations; SPSS: Statistical product and service solutions; SSC: Side scatter; TNF-α: Tumor necrosis factor-α; ULBP: UL16-binding protein.

## Competing interests

The authors declare that they have no competing interests.

## Authors’ contributions

Y-PP, YZ and J-JZ carried out the studies, participated in the statistical analysis and drafted the manuscript. Z-KX, Z-YQ, C-CD, K-RJ, J-LW, W-TG, QL and QD participated in the sample collection and statistical analysis. YM conceived of the study, and participated in its design and coordination and helped to draft the manuscript. All authors read and approved the final manuscript.
